# Treatment of Atlantoaxial Dislocation in Children with Down Syndrome Using Posterior Atlantoaxial Screw Fixation

**DOI:** 10.3389/fsurg.2022.877929

**Published:** 2022-05-26

**Authors:** Chengxin Li, Yiren Tian, Qiang Ren, Xiangqian Ji, Ziwei Mao, Ming Wu

**Affiliations:** ^1^ Department of Orthopedic, Beijing Children’s Hospital, Capital Medical University, Beijing, China; ^2^ Department of Orthopedic, Children’s Hospital of Hebei Province, Shijiazhuang, China

**Keywords:** down syndrome, children, os odontoideum, craniocervical junction instability, atlantoaxial dislocation

## Abstract

**Background:**

To investigate the effect of posterior atlantoaxial screw fixation for the treatment of atlantoaxial dislocation in children with Down syndrome (DS).

**Methods:**

Children diagnosed with DS who underwent posterior atlantoaxial screw fixation or occipitocervical fusion from January 2017 to January 2020 in Hebei Children’s Hospital were retrospectively included. Preoperative CT and MRI were performed to check the os odontoideum (OsO) and spinal cord compression, signal changes and spinal cord injury grade (ASIA grade).

**Results:**

All 5 children have atlantoaxial dislocation and OsO. Among which 60% (3/5) of children had changes in spinal cord signals and 40% (2/5) had dural sac compression. Every child underwent posterior atlantoaxial screw fixation (3.5-mm diameter), and the average fusion level was 1.8 (1–2). All 5 cases wore the head-neck-chest brace for 3–6 months after the operation. 1 case had dural tear and recovered well after timely suturing. 1 case had internal fixation breakage of the prosthetic joint and underwent revision surgery. At the last follow-up, all cases were fused and the neurological function were all ASIA grade E.

**Conclusion:**

After posterior atlantoaxial screw fixation, fusion and nerve recovery were achieved in all children with atlantoaxial dislocation and OsO. Postoperative head-neck-chest braces are necessary for children, especially those with occipitocervical fusion.

## Introduction

Down’s syndrome (DS), or trisomy 21, is a disorder caused by a chromosomal abnormality (an extra chromosome 21). Children with DS often have obvious growth retardation and multiple deformities (1). One-third of DS patients involve instability of the craniocervical junction (CVJ) (2), which is the main cervical spine problem in children with DS. Although CVJ instability is mostly asymptomatic (3, 4), the initial presentation of cervical instability can be catastrophic, often accompanied by respiratory insufficiency and potential risk of death.

Patients with DS are more prone to CVJ instability than the general population due to low bone density, inherent ligamentous laxity, high joint flexion, odontoid hypoplasia, and os odontoideum (OsO) (5, 6). Some children may develop atlantoaxial dislocation after minor trauma, resulting in severe spinal cord injury. Currently, the treatment of CVJ instability in children with DS, especially atlantoaxial dislocation, was mainly based on braces and halo external fixators, with poor treatment effects and many complications (7). However, there are few reports of internal fixation system therapy. Therefore, this study retrospectively analyzed and summarized the clinical data of 5 cases of atlantoaxial dislocation diagnosed with DS admitted to our hospital from 2017 to 2020, aiming to explore the clinical characteristics of atlantoaxial instability (AAI) or atlantoaxial dislocation (AAD) caused by pediatric DS and the effect of surgical treatment with posterior atlantoaxial screw fixation.

## Materials and Methods

### Patients

Five continuous children diagnosed with DS who underwent posterior atlantoaxial screw fixation or occipitocervical fusion from January 2017 to January 2020 in Hebei Children’s Hospital were retrospectively included. The inclusion criteria were children with the atlanto-dens interval (ADI) greater than 5 mm according to imaging examination. Children not followed up for more than 18 months (including revision cases) were excluded. This study was approved by the Medical research ethics committee of Hebei children’s Hospital (2021-88).

Preoperative CT and MRI were performed to check the OsO and spinal cord compression, signal changes and spinal cord injury grade (ASIA grade) (8).

### Treatment Method

Cranial traction were performed in five children intraoperatively using Gardner-Wells skull traction tongs (Qingniu Company, Suzhou, China). All five children underwent intraoperative cranial traction, nerve monitoring, and underwent posterior atlantoaxial screw fixation or occipitocervical fusion (9). The 3.5-mm titanium screws of the Occipito-Cervico-Thoracic System (Mountaineer OCT Spine System, DePuy Spine Inc., Raynham, MA, USA) were used. The screw length was determined according to the preoperative and intraoperative measurement results.

Briefly, after general anesthesia, the head is placed on a horse-shoe-shaped head support under the traction of the skull traction arch in the prone position, making the cervical spine to bend slightly and increasing the traction weight under the fluoroscopy of the c-arm, to observe the atlantoaxial position relationship and judge the effect of reduction. A median posterior cervical incision was made and subperiosteal dissection was performed. C2 screw placement and occipital plate fixation are both routines in children with DS. For C1 screw placement, the lateral mass is small, the operating space is limited, and the posterior arch of the atlas above the lateral mass is required. In the meantime, we should pay attention to protect the vertebral artery above the posterior arch, increase the space of the screw, then use the sharp cone to place the screw. On rare occasions, the vertebral artery may also enter the spinal canal beneath the posterior arch of C1 (the roatlantal variant type 2, and also the intradural vertebral artery variant). After the screw is placed, put the titanium rod, complete the reduction and carry out the autogenous bone graft.

More than 8 mL bone, mainly autologous bone, was taken for occipitocervical fixation. In occipitocervical fusion, the amount of bone graft is large, which is piled up and the stability after fusion is good.

All operations were performed by the same surgeon. No drainage was routinely placed. After the operation, the child can get out of bed after wearing a head-neck-chest brace.

### Follow-up and Clinical Evaluation

Follow-up was at least 18 months. Children were followed up at postoperative 3 months, 6 months, 1 year and once a year after surgery. Positive and lateral cervical spine X-rays and lateral hyperextension and flexion X-rays were performed during each follow-up. CT and MRI of the cervical spine were performed if necessary. The fixed segments, fusion and the recovery of neurological function were recorded.

## Results

The general information of 5 children are shown in Table 1. The average age was 4.2 years (3–5 years), and the average follow-up was 22 months (18–30 months). 3 cases (60%) had impaired neurological function (abnormal gait) and 2 cases (40%) had fixed/painful torticollis. All children have atlantoaxial dislocation. 5 cases underwent preoperative CT examination, and OsO was found in all 5 cases.

**Table 1:** Basic information, diagnosis and preoperative imaging of 5 children.

Cases	Gender	Age	Symptom	Diagnosis	ADI in neutral position (mm)	ADI in flexion position (mm)	ADI in extension position (mm)	Preoperative MRI	Preoperative CT	Preoperative ASIA grade
1	Female	4	Spinal cord injury, unsteady gait	Odontoid dysplasia, atlantoaxial dislocation, down’s syndrome, spinal cord injury	1.2	1.4	1.2	Hyperintense spinal cord edema	Os odontoideum	D
2	Female	3	Spinal cord injury, unable to stand or walk properly	Odontoid dysplasia, atlantoaxial dislocation, down’s syndrome, spinal cord injury, congenital heart disease	0.5	0.9	0.3	Hyperintense spinal cord edema	Os odontoideum	C
3	Female	5	Spinal cord injury, unable to stand or walk properly	Odontoid dysplasia, atlantoaxial dislocation, down’s syndrome, spinal cord injury	0.6	0.8	0.5	Hyperintense spinal cord edema	Os odontoideum	C
4	Female	4	Neck pain, limited movement	Odontoid dysplasia, atlantoaxial dislocation, down’s syndrome	0.8	1.0	0.7	Dural sac compression	Os odontoideum	E
5	Male	5	Neck pain, limited movement	Odontoid dysplasia, atlantoaxial dislocation, down’s syndrome	0.9	1.2	0.6	Dural sac compression	Os odontoideum	E

The preoperative average ADI was 10.6 mm (range 8–14 mm) in the flexion position, 8.0 mm (range 5–12 mm) in the neutral position, and 7 mm (range 3–12 mm) in the extension position. All children received preoperative MRI, 60% (3/5) showed changes in spinal cord signals, and 40% (2/5) showed dural sac compression. Every child underwent posterior atlantoaxial screw fixation (3.5-mm diameter), and the average fusion level was 1.8 (1–2). All 5 cases wore the head-neck-chest brace for 3–6 months after the operation.

**Figure 1:**
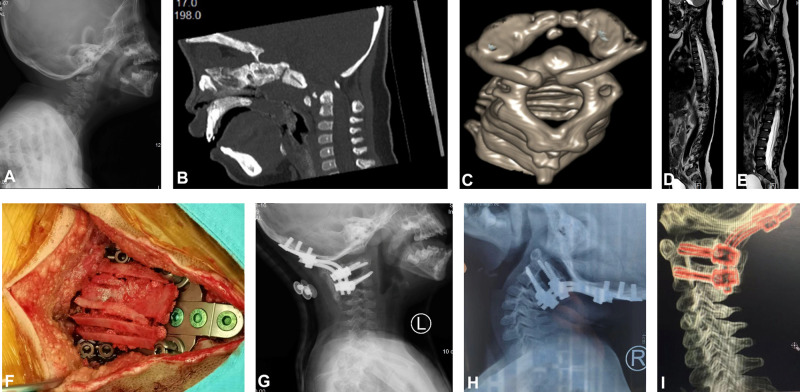
Case 2. A 3-year-old female child with DS, atlantoaxial dislocation, OsO, and spinal cord injury. (**A**) Dynamic X-ray showed that the ADI increased significantly, and a shift of the atlas anterior and lower to the axis. (**B**) Cervical sagittal reconstruction computed tomography (CT) and (**C**) three-dimensional reconstruction showed atlantoaxial dislocation with OsO. (**D** and **E**) Magnetic resonance imaging (MRI) showed that the upper cervical spinal cord was compressed by the upper posterior part of the odontoid process, and the spinal cord was edematous and degenerated. (**F**) Reduction was performed under cranial traction during surgery. (**G**) Postoperative X-ray films showed atlantoaxial screw rod fixation. (**H** and **I**) At postoperative 1 year, atlantoaxial CT and X-ray showed good atlantoaxial fusion and satisfactory internal fixation position.

**Figure 2:**
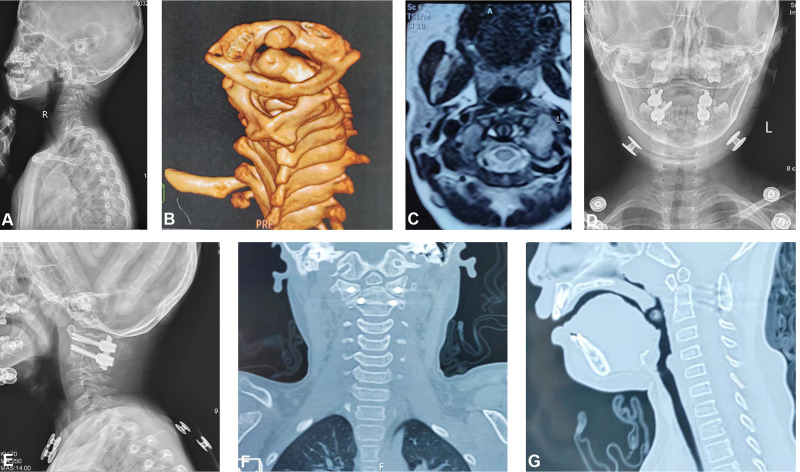
Case 4. A 4-year-old female child with DS, atlantoaxial dislocation with OsO. (**A**) Dynamic X-ray showed that the ADI increased significantly, and a shift of the atlas anterior and lower to the axis. (**B**) Three-dimensional reconstruction shows atlantoaxial dislocation with OsO. (**C**) MRI showed no compression of the upper cervical spinal cord. (**D** and **E**) Postoperative X-ray films showed atlantoaxial screw rod fixation. (**F** and **G**) At postoperative 1 year, atlantoaxial CT showed that the posterior atlantoaxial fusion was good, the internal fixation position was satisfactory, and there was no spinal canal stenosis.

Complications occurred in 2 cases. 1 case had dural tear between the posterior arch of the atlas and the lamina during the process of separation and exposure and recovered well after timely suturing. 1 case had internal fixation breakage of the prosthetic joint and underwent revision surgery. Posterior occipital fixation was performed again, with changed angle and direction of the screw, a new fixed pivot screw, and increased amount of bone graft. After enough brace wearing, fusion was finally obtained. No vertebral artery injury occurred in all cases. At the last follow-up, all cases were fused according to imaging examination results, and the neurological function were all ASIA grade E (Table 2). Two cases were shown in Figure 1 and Figure 2.

**Table 2:** Surgical method, fusion level, postoperative complications and fusion situation.

Cases	Fusion segment	Number of fusion segments	Fixation	Bone graft	External fixation	Fusion	Complication	Postoperative ADI	Duration of head-neck-chest brace (months)	Follow-up time (months)	Postoperative ASIA grade
1	Occipitocervical fixation (C0-C2)	2	C1,occipital plate; C2,4 screws	Autogenous bone	Yes	Yes	No	0.08	6	30	E
2	Occipitocervical fixation (C0-C2)	2	C1,occipital plate; C2,4 screws	Autogenous bone	Yes	Yes	Dural tear	0.08	4	24	E
3	Atlantoaxial fixation(C1-C2)	1	C1/C2,4 screws	Autogenous bone	Yes	Yes	No	0.10	3	20	E
4	Atlantoaxial fixation(C1-C2)	1	C1/C2,4 screws	Autogenous bone	Yes	Yes	No	0.08	3	19	E
5	Occipitocervical fixation (C0-C2)	2	C1,occipital plate; C2,2 screws	Autogenous bone	Yes	Fusion after revision surgery	Internal fixation breakage	0.29	6 + 6	18	E

## Discussion

OsO is the most common type of odontoid deformity (10), which is mostly accompanied by AAI, leading to atlantoaxial dislocation (AAD), spinal cord compression or paralysis. The etiology of OsO is still unclear, but the incidence of OsO is high in the DS population, especially in children and teenagers (11–13). In this study, all 5 children were accompanied by OSO and AAD.

In recent years, people have tried to apply the posterior cervical screw-rod system implantation for the treatment of atlantoaxial dislocation in DS patients. Although the sample size is limited, the results are encouraging. Ito et al. (14) used screw fixation to treat 2 DS patients with AAI, with improved neurological symptoms and good fusion. Yang et al. (15) reported 12 cases of CVJ instability or dislocation in DS patients with posterior cervical screw rod system fixation and achieved good results. Although nonunion occurred in 4 cases, there were no catastrophic complications such as death or quadriplegia. All 5 children in our study underwent posterior atlantoaxial screw fixation or occipitocervical fusion. 1 case had dural tear between the posterior arch of the atlas and the lamina during the process of separation and exposure and recovered well after timely suturing. 1 case had internal fixation breakage of the prosthetic joint and underwent revision surgery. Posterior occipital fixation was preformed again, with changed screw angle and direction, a new fixed pivot screw, and increased amount of bone graft. After enough brace wearing, fusion was finally obtained. This child had poor atlas development and large span of bone grafting area, and the child did not wear external fixation bracing postoperatively as required, which may be the reasons for the occurrence of bone nonunion. For children with DS, free odontoid ossicles and the discontinuity of the posterior arch of the atlas are prone to occur due to their poor coordination and inherent ligamentous laxity (16). Younger age, poor nutrition and bone quality, internal fixation firmness and holding force are all factors to consider when choosing a long-term firm external fixator after surgery. Therefore, for children with DS, we generally use head-neck-chest braces to ensure stability. We found that children with atlantoaxial fusion often get better fusion in 3 months, and children with occipitocervical fusion often need to extend the time of wearing the brace. Specifically, the external fixation support should be removed after the fusion effect has been confirmed by the review of cervical dynamic radiographs and atlantoaxial CT.

We found that early surgical treatment of atlantoaxial dislocation in children with DS has a high potential for neurological recovery. In our study, 3 out of 5 children had neurological symptoms before surgery. At the last follow-up, the neurological function ASIA grade of all children was grade E. Although dural sac compression and spinal cord edema can be observed in preoperative cervical spine MRI examination, our surgical strategy is mainly based on reduction and fusion, and we do not advocate indirect decompression. Since the AAD in most pediatric DS is reducible, we think additional decompressive surgery is not required after reduction.

In all cases, no preoperative traction was performed, but cranial traction was given under intraoperative anesthesia to evaluate the reduction of the atlantoaxial vertebrae. If it was reducible, posterior atlantoaxial screw fixation could be performed directly; if it was non-reducible, the anterior release was required (17). Previously, anterior decompression was limited to transoral release or odontoid excision, but several new approaches have emerged (18–20), some of which have been successfully applied in DS patients. Goel (18) adopted the techniques of posterior exposure of facet joint, joint surface separation and distraction, and reduction and internal fixation of atlantoaxial rotatory subluxation, which avoided the problems of infection and feeding in the anterior mouth release and achieved good results. Recently, transnasal endoscopic odontoidectomy has achieved good results in patients requiring odontoidectomy (19). We found that reactive soft tissue hyperplasia around the odontoid process, especially behind the odontoid process, is closely related to the progression of spinal cord compression. This reactive soft tissue may be cystic or fibrocartilaginous. Currently, the cause of these soft tissue hyperplasia is not well established. However, we found that the soft tissue of reactive hyperplasia around the odontoid was significantly reduced after fusion stabilization, and the spinal cord compression was significantly improved, indicating that anatomical reduction and stable fusion were more important than indirect decompression.

The following were some situations in which we think occipitocervical fixation (C0-C2) is needed: 1. Children with unstable atlanto-occipital joint (case 1); 2. For children younger than 4 years old, the inherent ligamentous laxity, poor bone quality, insufficient screw holding power, and the narrow operation space between the atlantoaxial vertebrae all hinders the sufficient and effective reduction operation. The choice of occipitocervical fixation has a relatively large operation space and a more accurate reduction effect (case 2); 3. Bone graft surface is lacking for children with dysplasia of the atlas, especially in the absence of the posterior arch. Moreover, the long-term dislocation of the atlantoaxial vertebra results in dysplasia of the lateral mass and concave in the articular surface, which is not conducive to the effective fixation of lateral mass screws. Therefore, occipitocervical fixation is selected to ensure the effect of bone graft fusion (case 2, case 5).

Previously, the evaluation of atlanto-occipital instability (AOI) was often based on Powers ratios, the Wiesel-Rothman technique, and Harris/BAI measurement, but all of them have their shortcomings. More recently, others have reported on the importance of mechanical deformity, described in terms of both the kyphosis of the cranio-cervical junction and the amount of pathological translation. The former is measured as the clival axial angle, or CXA (less than 135 degrees is potentially pathological) and the latter translation is measured from the Harris/BAI measurement in flexion subtracting the Harris measurement in extension: a translation of >4 mm is considered (21). In short, the assessment of AOI is very difficult, especially for children, since the assessment often requires CT and MRI examinations of the dynamic position. Children have a low degree of cooperation and often need to be performed under anesthesia, which make the clinical application difficult. Therefore, more feasible evaluation methods for AOI need to be further studied in the future.

CVJ instability is a major cervical spine problem in children with DS, which can cause trauma at any stage of fetal or child development. Therefore, regular screening may help clarify the cause and prevalence of odontoid abnormalities in DS patients and help prevent neurological deterioration. Screening can be accomplished by careful clinical examination and dynamic radiographs of the cervical spine in flexion and hyperextension (22). Some scholars (23) also recommended two screenings between the ages of 5–10 and 10–15 years old, respectively. MRI of CVJ in all children with DS can be a good choice for differentiating AAD from OsO. If AAI occurs, many authors recommend prophylactic surgery for all DS patients (24, 25). We suggest that children with DS should be screened regularly. Parents of children who have AAI should be educated, and some dangerous activities should be prohibited. If AAD occurs, surgery can be performed.

The small sample size and short follow-up were limitations in this study. Therefore, large randomized controlled trials remain to be done in the future.

## Conclusion

Posterior atlantoaxial screw fixation can stabilize the upper cervical spine for DS children with atlantoaxial dislocation, improve the compression of the spinal cord, and promote the recovery of nerve damage. In our study, fusion and nerve recovery were achieved in all children, and the results were very satisfactory. Postoperative head-neck-chest braces are necessary for children, especially those with occipitocervical fusion.

## Data Availability

The original contributions presented in the study are included in the article/supplementary material, further inquiries can be directed to the corresponding author/s.
